# Nanocomposite Hydrogels Based on Poly(vinyl alcohol) and Carbon Nanotubes for NIR-Light Triggered Drug Delivery

**DOI:** 10.1021/acsomega.3c09609

**Published:** 2024-02-26

**Authors:** Karla F. García Verdugo, Brianda M. Salazar Salas, Lerma Hanaiy Chan Chan, Dora E. Rodríguez Félix, Jesús M. Quiroz Castillo, Teresa del Castillo Castro

**Affiliations:** † Departamento de Investigación en Polímeros y Materiales, Universidad de Sonora, Hermosillo CP 83000, Mexico; ‡ Departamento de Física. Universidad de Sonora, Hermosillo, CP 83000, Mexico

## Abstract

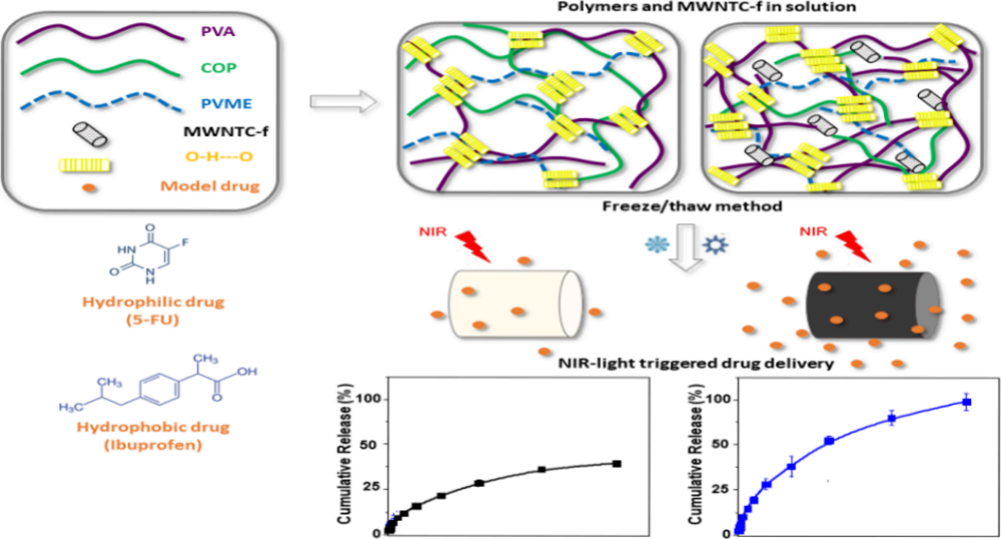

Photothermal nanocomposite hydrogels are promising materials for remotely triggering drug delivery by near-infrared (NIR) radiation stimuli. In this work, a novel hydrogel based on poly(vinyl alcohol), poly(vinyl methyl ether-*alt*-maleic acid), poly(vinyl methyl ether), and functionalized multiwalled carbon nanotubes (MWCNT-f) was prepared by the freeze/thaw method. A comparative characterization of materials (with and without MWCNT-f) was carried out by infrared spectroscopy, differential scanning calorimetry, scanning electron microscopy, mechanical assays, swelling kinetics measurements, and photothermal analysis under NIR irradiation. Hydrophilic chemotherapeutic 5-fluorouracil (5-FU) and hydrophobic ibuprofen drugs were independently loaded into hydrogels, and the drug release profiles were obtained under passive and NIR-irradiation conditions. The concentration-dependent cytotoxicity of materials was studied *in vitro* using noncancerous cells and cancer cells. Notable changes in the microstructure and physicochemical properties of hydrogels were observed by adding a low content (0.2 wt %) of MWCNT-f. The cumulative release amounts of 5-FU and ibuprofen from the hydrogel containing MWCNT-f were significantly increased by 21 and 39%, respectively, through the application of short-term NIR irradiation pulses. Appropriate concentrations of the nanocomposite hydrogel loaded with 5-FU produced cytotoxicity in cancer cells without affecting noncancerous cells. The overall properties of the MWCNT-f-containing hydrogel and its photothermal behavior make it an attractive material to promote the release of hydrophilic and hydrophobic drugs, depending on the treatment requirements.

## 1 Introduction

Stimuli-responsive hydrogels have shown relevant potential for biomedical applications. In this regard, composite hydrogels based on the combination of natural or synthetic polymers with nanofillers of different nature, such as metals, metal oxide, polymer, or carbonaceous materials, have been developed to fulfill the multiple requirements of some clinical uses.^1−4^ Particularly, hybrid hydrogels containing photothermal nanostructures have exhibited promising results for remotely triggered drug delivery by near-infrared (NIR) radiation stimuli.

Carbon nanotubes (CNT) have been extensively applied as an active filler for stimuli-responsive hydrogels. This well-studied nanomaterial exhibits outstanding properties including a high mechanical strength, large surface/volume ratio, high electrical conductivity, and thermal stability, as well as photothermal capabilities.^1,5,6^ In CNT, electrons can be promoted from the highest occupied molecular orbital (HOMO) energy level to the lowest unoccupied molecular orbital (LUMO) of higher energy upon irradiation in the NIR range. The relaxation from the excited state to the ground state occurs by electron–phonon coupling. The energy is transferred from the excited electrons to vibrational modes of the atomic lattices, producing an increase of material temperature.^7^ The hyperthermia effect of CNT embedded within a hydrated polymer network may promote structural transitions of the hydrogel, accelerate the chain motions, and change the diffusion rate of bioactive species loaded in the composite material, thereby intentionally modifying the kinetic of drug delivery.^6,8^

Different biodegradable and biocompatible polymers such as chitosan,^9^ polyethylene glycol,^10^ and poly(vinyl alcohol) (PVA)^11^ have been used as polymer matrix in CNT-containing nanocomposite hydrogels intended for biomedical applications. In this group, PVA stands out by its capacity to self-cross-link in aqueous solutions by the freeze/thaw method.^12,13^ This technique has proven effective to form stable physical hydrogels without using cross-linkers or comonomers that may induce cytotoxicity in a biological environment.^14,15^ In this regard, some works have proposed the use of PVA-based physical hydrogels containing CNT for controlled drug delivery applications. Huang et al. showed that polyvinylpyrrolidone (PVP)-wrapped CNT reinforced a physical PVA network as well as enhanced the electrical and thermal properties of hydrogels, evidencing their potential for a wide range of biomedical applications, including drug delivery.^11^ On the other hand, Özkahraman and Tamahkar reported an increase of 5-fluorouracil (5-FU) delivery with increasing the CNT content in nanocomposite hydrogels based on PVA and PVP obtained via the freeze/thaw method.^16^

The combination of PVA with polymers that contain hydrogen bond donor or acceptor groups has been used to strengthen the physical PVA networks via noncovalent interactions.^17−19^ Recently, our research group reported the preparation of semi-interpenetrating polymer networks formed by PVA, poly(vinyl methyl ether-*alt*-maleic acid) (COP in this report), and poly(vinyl methyl ether) (PVME) through an autoclaving process.^20^ COP is a water-soluble, biocompatible polymer that has been approved for biotechnology and pharmacology fields.^21−23^ On the other hand, PVME has been used as a steric stabilizer in several polymer blends due to its dual hydrophilic and hydrophobic nature.^24−26^ In this sense, the design and preparation of multifunctional composite hydrogels by simple and nontoxic methods are important goals toward the construction of NIR light-responsive platforms for biomedical applications.

In this work, novel hydrogels based on PVA, COP, and PVME, with and without functionalized multiwalled carbon nanotubes (MWCNT-f), were prepared by the freeze/thaw method. The morphological, structural, and physicochemical properties of the polymer hydrogel (H-polymer) and those of the nanocomposite hydrogel (H-MWCNT-f) were assessed. The photothermal behavior of the hybrid hydrogels was evaluated under NIR radiation. Moreover, the *in vitro* release of 5-FU and ibuprofen, as model hydrophilic and hydrophobic drugs, respectively, was studied under passive and NIR radiation-stimulated conditions. Finally, the concentration-dependent cytotoxicity of the hydrogels and the biological activity of the 5-FU were assessed via a resazurin reduction assay using noncancerous and cancer cells.

## 2 Experimental Section

### 2.1 Materials

Poly(vinyl alcohol) (PVA) Mw 85,000–124,000, 99% hydrolyzed; poly(vinyl methyl ether-*alt*-maleic anhydride) (PVME-MA) Mw 216,000; poly(vinyl methyl ether) (PVME) 50 wt % in water; multiwalled carbon nanotubes (MWCNT) (≥98%, Aldrich, OD × ID × *L*: 10 ± 1 nm × 4.5 ± 0.5 nm × 3–6 μm); sodium dodecyl sulfate (SDS); 5-fluorouracil (5-FU) 99%; and ibuprofen sodium salt ≥98% were purchased from Sigma-Aldrich. The PVME-MA reagent was dissolved in water and thermally treated at 70 °C for 6 h to obtain poly(vinyl methyl ether-*alt*-maleic acid) (COP).^20^ Functionalized multiwalled carbon nanotubes (MWCNT-f) were obtained by the microwave-assisted treatment of MWCNT with a strong acid mixture, as reported previously by our research group.^27^ In that work, TEM images revealed that the nanotubes preserved their integrity without severe changes in their surface morphology and length. All aqueous solutions were prepared with deionized water (DI), which was obtained by a Milli-Q Organex system (Millipore).

### 2.2 Synthesis of Hydrogels

The freeze–thaw method was used to prepare two hydrogel formulations: (1) a hydrogel containing PVA, COP, and PVME (H-polymer) and (2) a nanocomposite hydrogel based on PVA, COP, PVME, and MWCNT-f (H-MWCNT-f). Aqueous solutions of 7.5 wt % PVA, 15.0 wt % COP, and 50.0 wt % PVME were used as starting solutions. These solutions were properly mixed to achieve a PVA/COP/PVME mass ratio of 55/34/10. Portions of 2 mL were poured into cylindrical molds of 10 mm diameter × 20 mm height and frozen at −14 °C for 18 h. Then, the solid samples were thawed at room temperature (∼25 °C) for 6 h. Two freeze–thaw cycles were used to form the hydrogels. In the case of H-MWCNT-f hydrogels, before the freeze–thaw process, the required amount of MWCNT-f was sonicated in a 30 mM SDS solution for 30 min, and the resultant suspension was mixed with the polymer solution to reach 0.2 wt % of nanotubes in the final material. As-formed hydrogels were freeze-dried using a Labconco FreeZone 4.5 L vacuum freeze-dryer for the following procedures.

### 2.3 Characterization of Hydrogels

Fourier transform infrared spectroscopy (FTIR) analysis was carried out in a PerkinElmer equipment model Frontier in the 4000 to 400 cm^–1^ range using the KBr technique. Thermal properties were evaluated by differential scanning calorimetry (DSC) in a PerkinElmer DSC 8500 equipment, under a nitrogen atmosphere, at a 10 °C min^–1^ heating rate. Morphology was studied by scanning electron microscopy (SEM) using a JEOL JSM-7800F microscope. The mean pore size was calculated by the software ImageJ using the SEM images. Samples were cut in a lamellar way, quickly frozen in liquid nitrogen, and lyophilized on a freeze-dryer Labconco FreeZone 4.5 L. The dried samples were fixed on carbon ribbon and gold sputtered prior to their analysis. Mechanical properties of hydrated hydrogels were evaluated in compression tests using a TA ElectroForce 5500 BioDynamic equipment with a 200 N load cell. Cylindrical-shaped hydrogels of 10 mm diameter × 15 mm height were compressed at a constant strain rate of 0.1 mm s^–1^. Young’s moduli were determined from the first section of the J-shaped stress–strain curve up to 10% strain. Swelling kinetics of hydrogels was obtained by the gravimetric method in a PBS buffer (pH 7.4) solution using a Precision 2870 bath at controlled temperatures of 25 and 37 °C. The swelling percentage was calculated by the formula
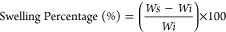
1where *W*_s_ is the weight of the swollen gel over time until equilibrium and *W*_i_ is its initial weight.

### 2.4 Photothermal Effect Measurement

H-polymer and H-MWCNT-f hydrogels, previously hydrated for 4 h in the PBS buffer (pH 7.4), were irradiated with an NIR laser (Opto Engine, model PSU-III.LED, 808 nm) for 10 min at a radiation power of 1 W cm^–2^. During irradiation, the temperature of hydrogels was measured by using a thermocouple probe inserted in the hydrogel and connected to an Agilent multimeter model 34410A. Data were processed by the Agilent IntuiLink software. Thermal images of hydrogels were also acquired with an FLIR E53 thermographic camera.

### 2.5 Load and *In Vitro* Release Study of 5-FU and Ibuprofen from Hydrogels

5-FU and ibuprofen were used as model drugs to study their release kinetics from hydrogels with and without NIR irradiation. Total amounts of 4 and 5.3 mg of 5-FU and ibuprofen, respectively, were loaded into the hydrogels during their preparation. Drug portions were added to the COP/PVME solution with stirring for 2 h. Then, the PVA solution and, in the case of the H-MWCNT-f hydrogel, the MWCNT-f amount were added to the precursor mixture to form the drug-loaded hydrogels by the freeze-thaw method, as previously described in Subsection 2.2. All experiments of 5-FU and ibuprofen release were conducted in 100 mL of the PBS buffer (pH 7.4) at a controlled medium temperature of 37 °C. At specific time intervals, 1 mL of the release medium was withdrawn and replaced with 1 mL of fresh medium. The concentrations of 5-FU and ibuprofen were determined by UV–vis absorption spectroscopy at 266 and 223 nm, respectively, on an Agilent 8435 spectrophotometer. In the case of optically stimulated experiments, the drug-loaded hydrogels with an inserted thermocouple probe were immersed in the release medium. For the 5-FU release, hydrogel samples were irradiated with 3 min lasting NIR light (808 nm) of 1 W cm^–2^ at specific times of 64 and 194 min. On the other hand, a higher time and light power of 5 min lasting NIR light (808 nm) of 2 W cm^–2^ was used for the ibuprofen release study, with irradiations at 460 and 1600 min.

### 2.6 Cell Culture and Cytotoxicity Assays

The *in vitro* cytotoxicity assay of hydrogel samples was performed on noncancerous and cancer cells by the indirect contact method. Tests in triplicate were used in different hydrogel formulations according to ISO 10993-5-2009 guidelines. A noncancerous mouse fibroblast cell line derived from normal subcutaneous connective tissues (L-929, NCTC clone 929 from ATCC) and, alternatively, human cervical cancer cells (HeLa obtained from ATCC) were cultured in Dulbecco’s modified Eagle medium (DMEM, CAISSON Laboratories) supplemented with 5% heat-inactivated fetal bovine serum (Gibco) and 1% of penicillin/streptomycin solution (10,000 IU penicillin + 10 mg streptomycin/mL, Sigma-Aldrich) in a controlled atmosphere (80–90% humidity, 37 °C, 5% CO_2_). Hydrogel samples (330 μL), previously sterilized with UV light for 15 min each side, were incubated in 4 mL of the supplemented culture medium at 37 °C for 24 h. Then, 2 mL of the conditioned medium (extract) was filtered with a 0.2 μm syringe filter (Corning) and preserved for the viability assays. Different concentrations were obtained by serial dilution of the extracts in the supplemented culture medium and used in the experiments as follows. First, the cells were seeded in a 96-well plate at a density of 5000 cells/well. After 24 h of incubation, the culture medium was removed, and conditioned media at different concentrations were added to each well by triplicate. In the wells of the positive control, the culture medium was added to the cells instead of extract solutions. Wells without cells were used as the negative control. At 24, 48, and 72 h, cellular viability and cytotoxicity were determined by measuring the reduction of resazurin.^28^ The concentrated stock solution (4 mg/mL) of resazurin salt (Sigma-Aldrich) was diluted in the supplemented culture medium and added to each well at a final proportion 1:200. After 6 h of incubation, the absorbance of the samples was measured at 570 and 600 nm using a Synergy HTX multimode microplate reader (BioTek Instruments, Vermont, USA.). Viability percentages were calculated by the formula of Zapata-Catzin et al.^29^

### 2.7 Statistical Analysis

Results are presented as the mean ± standard deviation. Statistical analysis was performed by means of one-way analysis of variance (ANOVA) with the NCSS software. A difference of *p* < 0.05 was considered statistically significant.

## 3 Results and Discussion

### 3.1 Hydrogel Formation

**Figure 1:**
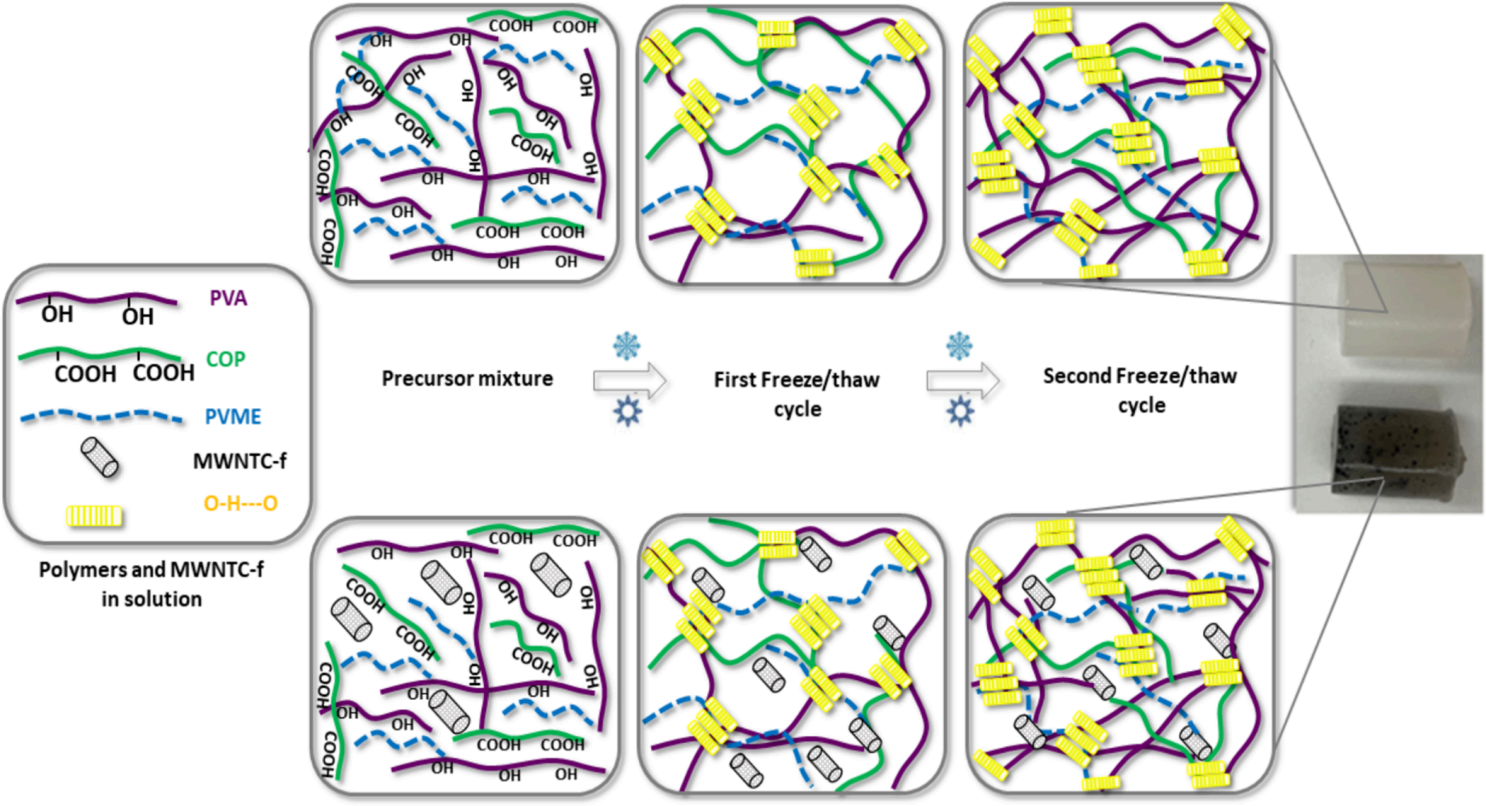
Schematic representation of the preparation of physically cross-linked hydrogels by the freeze/thaw method. Images of as-prepared H-polymer (whitish hydrogel) and H-MWCNT-f (dark hydrogel) samples are included (the photograph was taken by the authors).

Physically cross-linked hydrogels based on polymers PVA, COP, and PVME were successfully synthesized. The multicomponent networks were built by the phase separation of the PVA solution during the freeze–thaw method (Figure 1). It has been well-known that the phase separation of the PVA solution occurs at water-freezing conditions, leading to a polymer-rich phase in which the close interchain contact promotes intermolecular hydrogen bonding and crystallite formation. The crystalline regions remain intact after samples are thawed at room temperature, and the three-dimensional polymer network is preserved.^30^

It was expected that the incorporation of COP and PVME into the cross-linked PVA matrix can reinforce the three-dimensional structure by physical interaction between carboxyl groups (COOH) of COP, ether groups (C–O–C) of PVME, and hydroxyl groups (OH) of PVA.^31^ In the case of the nanocomposite hydrogel, the MWNTC-f were able to participate in the supramolecular polymer architecture through hydrophobic interactions and also forming hydrogen bonds mediated by oxygen groups of their surfaces. Figure 1 represents the possible interactions between components of the H-MWCNT-f hydrogel formed by the freeze–thaw method.

In this work, cylindrical-shaped hydrogels of 10 mm diameter × 15 mm height were obtained; however, H-polymer and H-MWCNT-f hydrogels of different geometries can also be prepared by the same preparation method. The dispersion of carbonaceous nanomaterials within a hydrophilic environment has been a challenge, especially when they are subjected to freezing conditions. The CNT tend to form bundles, entanglements, and agglomerates due to their intrinsic van der Waal interactions.^11,32^ By the naked eye, regions of high concentration of nanotubes were distinguished on the surface of gray-colored H-MWCNT-f hydrogels. In this work, the lowest possible concentration of the SDS surfactant (1.25 mM) was used to minimize the aggregative tendency of MWCNT-f during the freeze–thaw cycles.

### 3.2 Characterization

**Figure 2:**
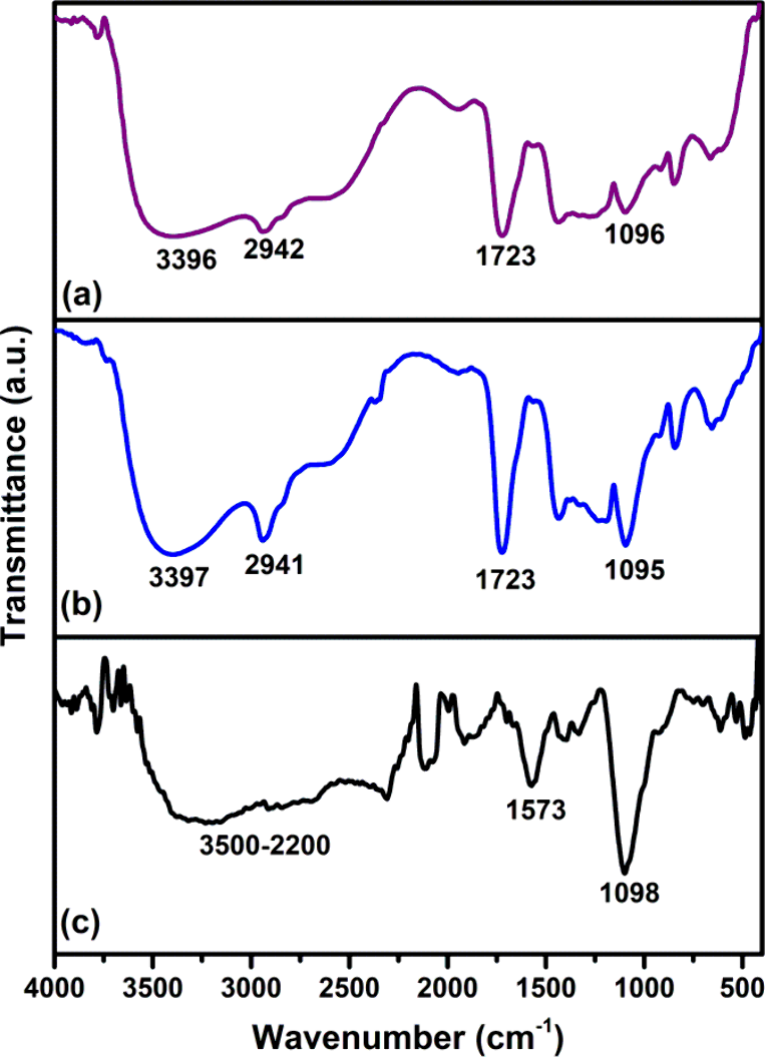
FTIR spectra of the H-polymer hydrogel (a), H-MWCNT-f hydrogel (b), and MWCNT-f filler (c).

FTIR was used to gain insight into the degree of polymer–polymer and polymer–nanotube interactions in the hydrogel. Figure 2 shows the FTIR spectra of the H-polymer hydrogel, as well as those of the MWCNT-f sample and the H- MWCNT-f hydrogel.

The H-polymer spectrum exhibits the main bands of neat polymers (Figure 2a).^20^ The band at 3396 cm^–1^, broadened toward lower wavenumbers, is associated with the stretching vibration of hydrogen-bonded OH groups of PVA and COP. During the freeze–thaw process, the side OH groups of PVA form long-range intermolecular hydrogen bonds between polymer chains, leading to the physically cross-linked PVA network.^31^ As mentioned earlier, carboxyl groups of COP and ether groups of PVME are able to form further hydrogen bonds with PVA, reinforcing the polymer network.^33^ The signal located at 2942 cm^–1^ corresponds to the stretching vibration of the C–H bond.^34,35^ The strong peak at 1723 cm^–1^ is attributed to the stretching vibrations of carbonyl groups (C=O) of COP.^36^ The peak at 1096 cm^–1^ is attributed to the stretching vibration of the C–O bond and the bending mode of the ether group (OCH_3_) of both COP and PVME units.^37^

The MWCNT-f spectrum exhibits the typical bands of functionalized carbon nanomaterials (Figure 2c). A broad band attributed to the stretching vibration of the O–H groups appears in the 3500–2200 cm^–1^ region due to the carboxyl group contribution of nanotube surfaces. The band at 1573 cm^–1^ is related with the stretching vibration of the C=C bond,^38^ whereas the signal corresponding to the stretching vibration of the C–O bond is observed at 1098 cm^–1^.^27^

Figure 2b shows the spectrum of the H-MWCNT-f hydrogel that exhibits slight differences as compared with that of the H-polymer hydrogel. The absorption corresponding to the O–H bond (3397 cm^–1^) is narrower in the H-MWCNT-f spectrum as compared to that of the H-polymer hydrogel. This spectral feature suggests that the polar groups of MWCNT-f surfaces modified the electronic environment of OH moieties of polymers. Furthermore, the relative intensity of the peak attributed to stretching vibration C–O bond (1095 cm^–1^) is higher in the nanotube-containing hydrogel than in the H-polymer sample due to the overlapped contribution of COP, PVME, and MWCNT-f.

**Figure 3:**
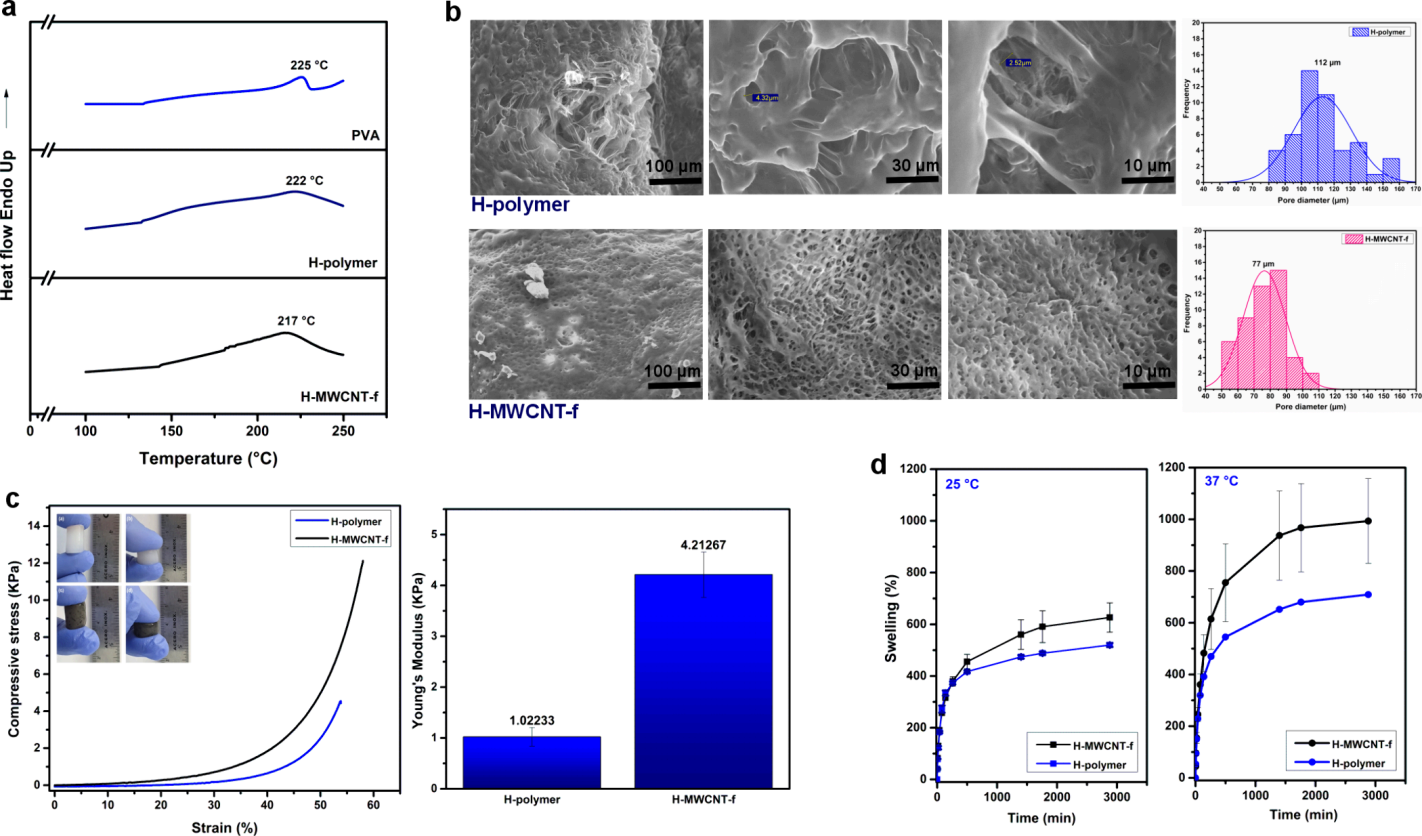
DSC curves of the PVA polymer and those of the H-polymer and H-MWCNT-f hydrogels (a). SEM images and pore size distribution histograms of freeze-dried hydrogels (b). Stress–strain curves, Young’s modulus, and representative images of the compression behavior of materials (c). Swelling measurements of hydrogels in PBS pH 7.4 at 25 and 37 °C (the photographs were taken by the authors).

DSC analysis was performed to evaluate the physicochemical modification of PVA during the synthesis of hydrogels. Figure 3a shows the DSC curves of linear PVA and those of the H-polymer and H-MWCNT-f hydrogels. The thermal curve of PVA showed an endothermic signal at 225 °C corresponding to the melting temperature (*T*_m_) of polymer. Similar *T*_m_ values have been reported for 99% hydrolyzed PVA samples.^31,39^

On the other hand, no-well-defined melting events appear at lower temperatures for the hydrogels, particularly in the nanocomposite hydrogel (*T*_m_ = 217 °C), as compared to the neat-PVA curve. This result confirmed that the carbonaceous nanofiller interacts at the molecular level with the polymer network, thereby modifying the crystallinity grade of PVA in the nanocomposite hydrogel.

Figure 3b shows SEM micrographs of the cross sections of the H-polymer and H-MWCNT-f hydrogels. Both samples exhibited a highly porous morphology; the mean pore sizes were found to be 112 ± 17.84 and 77 ± 13.09 μm for the H-polymer and H- MWCNT-f hydrogel, respectively. It is suggested that the reduction of the pore size of hydrogels by adding the MWCNT-f was associated with the physical interactions between the carbonaceous nanofiller and the polymer network at the molecular level. This result is consistent with DSC analysis and indicated that the polymer–MWNTC-f interaction promoted the microstructural homogeneity of the material.^40^ In a previous report, Xu and Li prepared composite hydrogels of PVA filled with cellulose nanofibers (CNF) by the freeze/thaw method. The pore sizes of these highly porous hydrogels were controlled by the CNF addition.^41^

Figure 3c depicts the stress–strain curves and Young’s modulus results of compression tests of H-polymer and H-MWCNT-f hydrogels. Both hydrogels exhibited a J-shaped stress–strain behavior that it is typical of load-bearing soft tissues, with a high compliance at low strains and a high strength at high strains.^42^ The H-MWCNT-f hydrogel showed a higher strength in all deformation ranges than the H-polymer hydrogel. Indeed, the Young’s modulus of the nanocomposite hydrogel with only 0.2 wt % of MWCNT-f was 4 times higher than that of the nanotube-free hydrogel.

The mechanical strength of hydrogels directly depends on the microstructural morphology, the cross-linking degree, and the size porosity of the material.^41^ According to SEM micrographs, the H-MWCNT-f hydrogel showed smaller pore sizes in comparison to the H-polymer hydrogel. The pore size reduction and a uniform pore distribution typically increase the rigidity of hydrogels due to a better load distribution throughout the cross-linking points.^43^ On the other hand, it has been well-known that the inherent high mechanical strength of the carbonaceous nanomaterials promotes the reinforcing of composite materials.^44^ This effect is particularly useful in physically cross-linked hydrogels such as those prepared in the present work. Similar works that prepared hybrid hydrogels based on PVA and CNT by the freeze/thaw method have reported an improvement of the mechanical strength of materials by adding the carbonaceous filler.^45,46^

Figure 3d shows the swelling profiles of H-polymer and H-MWCNT-f hydrogels at temperatures of 25 and 37 °C in PBS buffer, pH 7.4. At both temperatures, the H-MWCNT-f hydrogel exhibits a higher equilibrium swelling level (626% at 25 °C and 993% at 37 °C) as compared to the H-polymer hydrogel (520% at 25 °C and 709% at 37 °C). This swelling behavior is in good agreement with the previously discussed results of FTIR, DSC, and SEM techniques. MWCNT-f hindered the PVA crystallization and produced a uniform porous microstructure, promoting the water uptake.^41^ As expected, the swelling capacity of both hydrogels was higher at 37 °C in comparison with the swelling percentage at 25 °C due to kinetic contributions.^15^

### 3.3 Photothermal Effect of the H-MWCNT-f

Previous findings have shown the NIR absorption capabilities and the photothermal behavior of MWCNT-based materials.^6,8,47^ Indeed, MWCNT-containing hydrogels have been explored with positive results in photothermal therapies and for controlled drug delivery activated by NIR radiation.^8,48^ In this work, the thermal response of hydrogels with and without MWCNT-f was investigated by irradiating the samples with an NIR laser of 808 nm at 1 W cm^–2^ power for 10 min.

**Figure 4:**
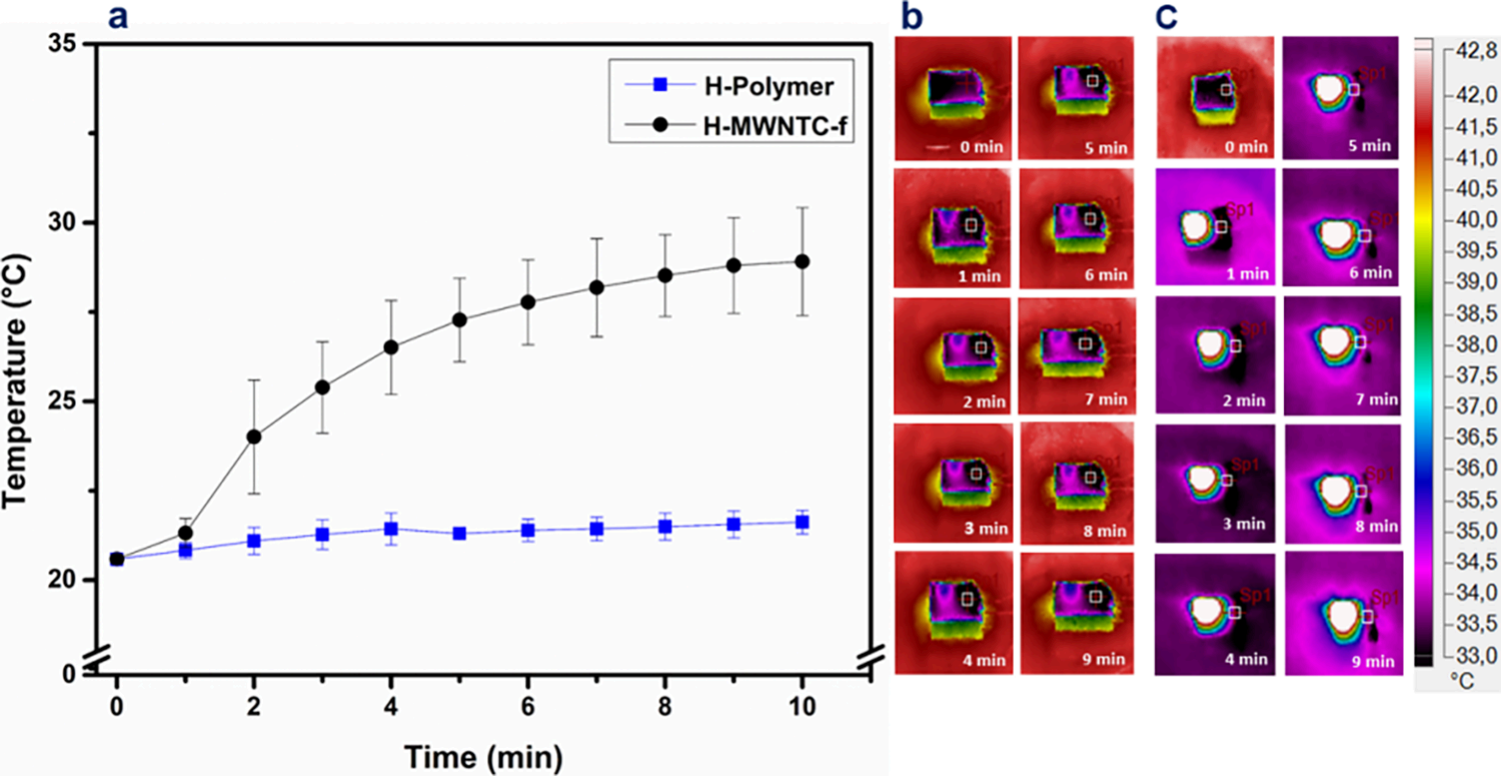
Temperature profiles of H-polymer and H-MWCNT-f hydrogels, previously swollen in PBS buffer (pH 7.4), under NIR laser irradiation (808 nm, 1 W cm^–2^) (a). Representative thermographic images of H-polymer (b) and H-MWCNT-f (c) samples during irradiation.

Figure 4a displays the profiles of the internal temperature as a function of irradiation time for both hydrogels in a relaxed state. A slight increment of 1 °C was observed for the H-polymer hydrogel after 10 min of laser irradiation. Conversely, the nanocomposite hydrogel increased its internal temperature up to 9 °C with respect to its initial temperature. These results demonstrated that the nanocomposite hydrogel was able to convert NIR light into thermal energy, in accordance with the typical photothermal behavior of its carbonaceous filler. Thermal images of H-polymer (Figure 4b) and H-MWCNT-f (Figure 4c) hydrogels illustrated the different evolution stages of the external temperature for both materials, confirming the photothermal response of the H-MWCNT-f sample under NIR light stimulus.^49,50^ Similar results were reported by Dong et al., who observed a temperature increment of 15 °C for a composite hydrogel based on polycaprolactone (PCL)-poly(ethylene glycol)-PCL and MWCNT by NIR irradiation.^8^

### 3.4 *In Vitro* Drug Release Study of 5-FU and Ibuprofen

Specific drug release behaviors are required according to the principles of each clinical treatment. Stimuli-responsive hydrogels have shown some benefits in the drug delivery field.^2^ External stimulus can be used to accelerate or retard the drug release kinetics, increase the amount of drug release at the target site, and induce pulsatile drug release profiles, among other desired effects. In this work, 5-FU and ibuprofen were independently loaded into H-polymer and H-MWCNT-f hydrogels to evaluate the effect of NIR irradiation on the drug release kinetics.

**Figure 5:**
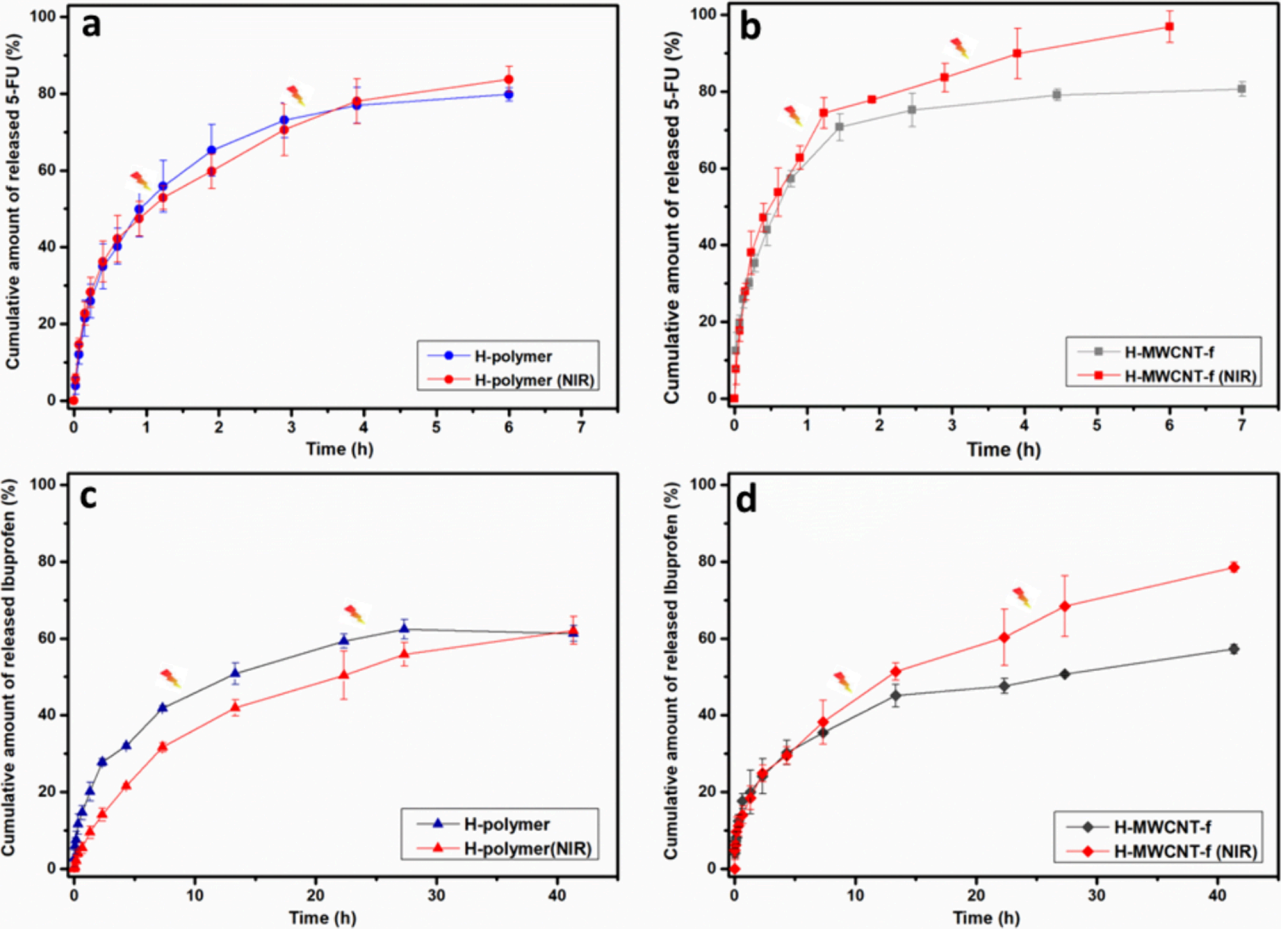
Release profiles of 5-FU (a, b) and ibuprofen (c, d) from H-polymer and H-MWCNT-f hydrogels in PBS buffer (pH 7.4) at 37 °C with and without NIR light stimuli.

Figure 5 displays the *in vitro* release profiles of 5-FU and ibuprofen from hydrogels under passive conditions and with the application of pulse NIR radiation. The conditions of shorter irradiation time and lower irradiation power were established under which significant changes in drug release kinetics occurred with respect to passive administration.

The 5-FU release profiles (Figure 5a,b) showed a burst release in the first 1.5 h for both hydrogels, achieving 80% of drug release at equilibrium within the 6 h of passive delivery. 5-FU is a low-molecular-weight, hydrophilic drug that it is rapidly released from its carrier matrix.^51,52^ A portion (∼20%) of 5-FU guest molecules was retained within the H-polymer and H-MWCNT-f hydrogels due presumably to supramolecular interactions between the heterocyclic aromatic drug and the chemical moieties of hydrogel components. No significant changes in the equilibrium drug release were observed when the H-polymer hydrogel was irradiated with two pulses of 3 min NIR light in comparison with the unstimulated drug release experiment (Figure 5a). In contrast, the 5-FU release kinetics of the H-MWCNT-f hydrogel was activated when the sample was irradiated for 3 min twice with NIR light, significantly increasing (*p* < 0.05) the cumulative amount of equilibrium drug release up to 97% as compared to the value of 80% in passive conditions (Figure 5b). The activation of drug release in the nanocomposite hydrogel was attributed to the photothermal effect of MWCNT-f. The increase of the internal temperature of the H-MWCNT-f hydrogel due to the NIR irradiation, increased the chains mobility and the diffusion rate of the drug through and out of the material, promoting an almost complete release of the 5-FU contained in the hydrogel.

On the other hand, both hydrogels exhibited a sustained release of ibuprofen up to almost 47 h (Figure 5c,d). The cumulative release amounts of ibuprofen at equilibrium were 61 and 57% for H-polymer and H-MWCNT-f hydrogels, respectively, in passive delivery. The H-polymer hydrogel did not show significant changes in the amount of drug release at equilibrium when NIR light was applied. Instead, the ibuprofen delivery significantly increased (*p* < 0.05) up to 79% with the application of two pulses of 5 min NIR light.

These results confirmed the photothermal capacity of the H-MWCNT-f hydrogel and the positive effect of NIR radiation to promote the release of hydrophilic and hydrophobic drugs. The nanocomposite hydrogel was able to partially retain amounts of 5-FU and ibuprofen and release them in response to different NIR light power and time irradiation. Similar conditions have been used in the literature for the application of optical stimuli in biomedical applications, without reporting tissue damage.^53^ In this manner, the NIR-mediated photothermal effect can be used to remotely control the on-demand release of these drugs depending on dosage and treatment.

### 3.5 Cytotoxicity of the H-MWCNT-f Hydrogel

Materials based on CNT have been extensively explored for biomedical applications. Nevertheless, information about the biocompatibility and toxicity of the CNT in different biological environments has been controversial. Several studies have reported that systems containing MWCNT-f are innocuous, whereas other works have concluded that the carbonaceous materials are cytotoxic for certain tissues and cells.^54−56^ Overall, CNT toxicity depends on their concentration, surface area, shape, and size.^57^ In this work, an *in vitro* resazurin reduction assay was performed to evaluate the concentration-dependent cytotoxicity of the hydrogels with and without MWCNT-f.

**Figure 6:**
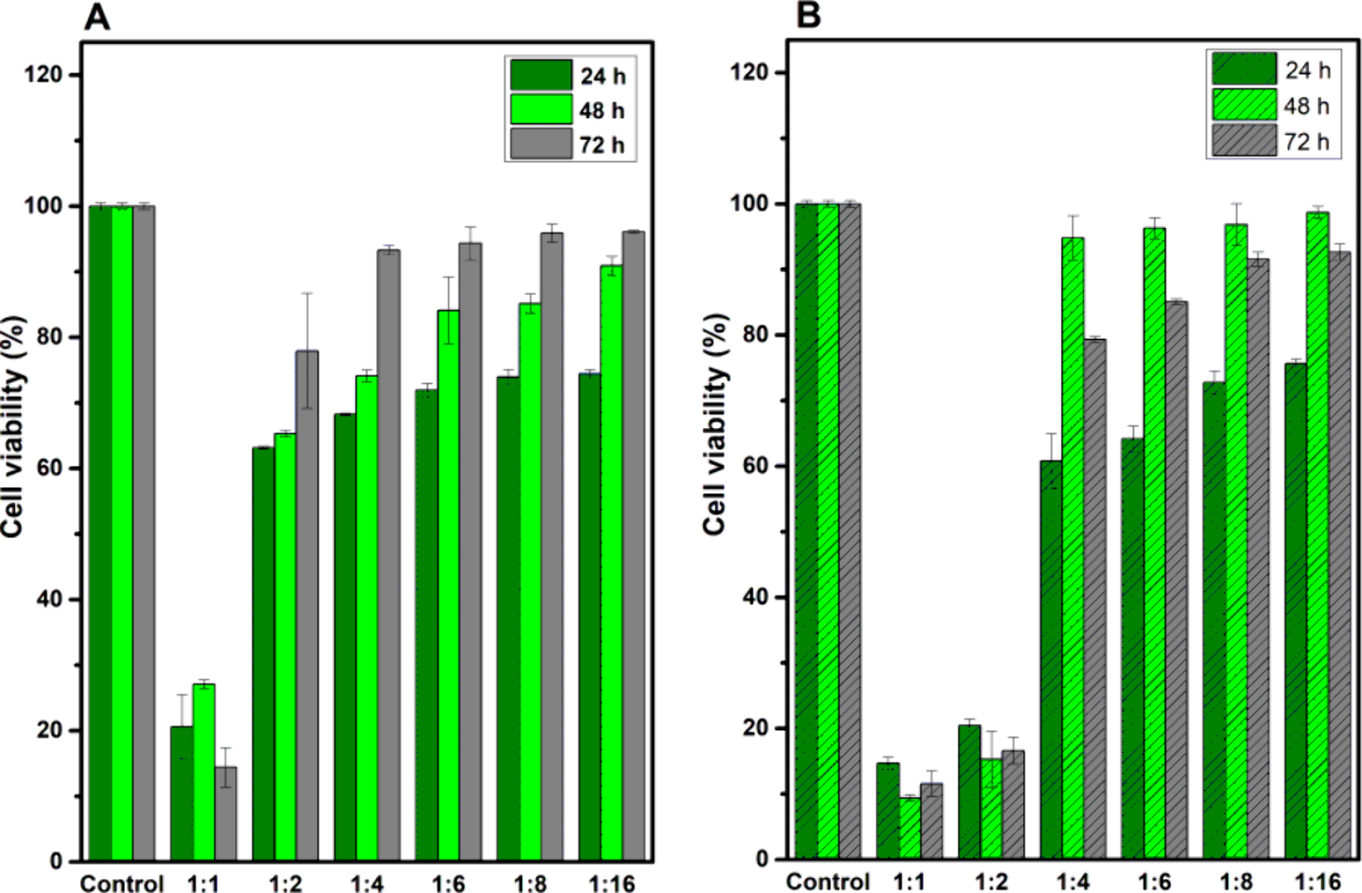
Viability of L-929 cells exposed to different concentrations of conditioned media from H-polymer (A) and H-MWCNT-f (B) hydrogels without drugs. Data are shown as the mean ± SD from three independent repeats at different times in the cell culture.

Figure 6 exhibits the viability results of L-929 cells exposed to different dilutions of H-polymer and H-MWCNT-f conditioned media without the drug model. The H-polymer hydrogel did not show a toxicity effect at dilutions of conditioned media from 1:4 to 1:16, in which cell viabilities were higher than 80% (Figure 6a) according to the toxicity minimal value established by ISO 10993-5-2009. Similarly, a dose-dependent effect on cell viability was observed for conditioned medium with the H-MWCNT-f sample, finding a marked decrease of viability when noncancerous cells were exposed to extracts of 1:2 and 1:1 dilution (Figure 6b). These results evidenced that hydrogels induce cytotoxicity of L-929 cells at high concentration levels.

**Figure 7:**
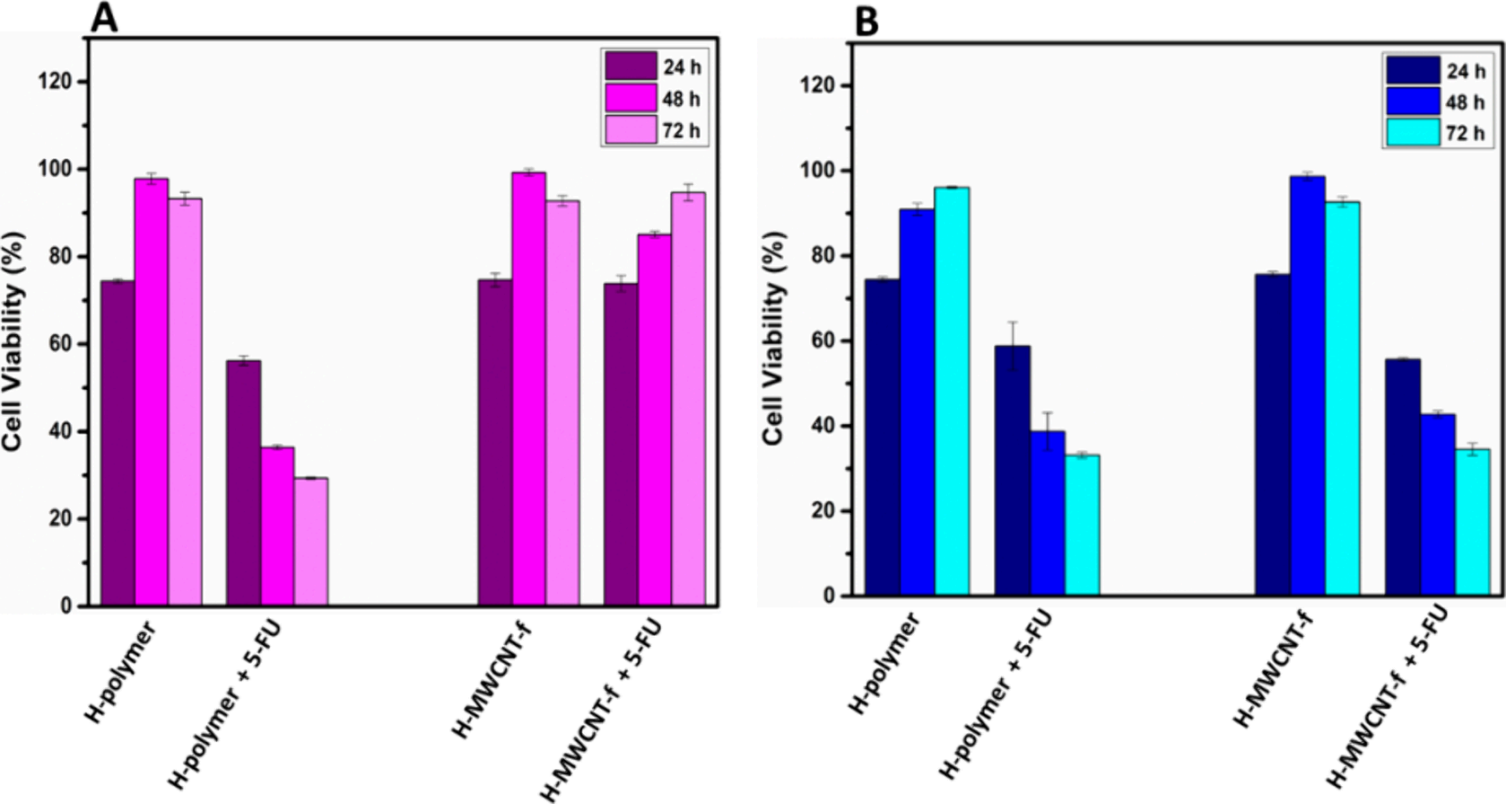
Viability of L-929 cells (A) and HeLa cancer cells (B) exposed to 1:16 dilution of conditioned media from H-polymer, H-polymer containing 5-FU, H-MWCNT-f, and H-MWCNT-f containing 5-FU hydrogels. Data are shown as the mean ± SD from three independent repeats at different times of cell culture.

The biological activity of 5-FU released from hydrogels was assessed via a resazurin reduction assay using L-929 cells and HeLa cancer cells. The cells were exposed to extracts obtained from neat hydrogels and 5-FU-containing hydrogels of the lowest dilution (1:16). The extract of the H-polymer hydrogel containing 5-FU produced a significant decrease of viability of noncancerous cells (Figure 7a) and cancer cells (Figure 7b). Several studies have reported that 5-FU affects the morphology and the cycles of proliferation of both types of cells, inducing apoptosis.^58,59^ The extract of H-MWCNT-f hydrogel loaded with 5-FU induced a significant decrease of the viability of cancer cells, whereas 93% cell viability was observed for noncancerous cells after 72 h. It is suggested that some interactions between nanotubes and 5-FU resulted in a lower drug availability in the conditioned media exposed to the H-MWCNT-f hydrogel, leading to a higher viability of noncancerous cells (nontoxic dose). This effect was not observed for cancer cells, evidencing a higher sensitivity of these cells to the concentration of 5-FU released from both hydrogels to the conditioned medium. Based on these observations, the nanocomposite hydrogel may be a promising drug delivery system in cancer treatment without compromising noncancerous cells using appropriate concentrations of the nanomaterials and drug doses.

## 4 Conclusions

Structurally uniform novel hydrogels based on PVA, COP, PVME, and MWCNT-f were successfully synthesized by a freeze/thaw process. The preparation method allowed obtaining a composite material in the absence of toxic monomers, initiators, and cross-linkers, which is an advantage for biomedical applications. The physicochemical properties of the nanocomposite hydrogel differed from those of the filler-free hydrogel. MWCNT-f were able to interact at the molecular level with the polymer network, modifying the microstructure of material and thereby producing notable changes in its morphology, swelling behavior, and mechanical strength. The photothermal capability of the H-MWCNT-f encapsulated within the hydrogel allowed the activation of the release of 5-FU and ibuprofen by short-term NIR stimuli, significantly increasing the maximum amount of drug released from the nanocomposite hydrogel as compared to the drug delivery in passive conditions. Biological evaluation of hydrogels evidenced that appropriate concentrations of the nanocomposite hydrogel loaded with 5-FU produced cytotoxicity in cancer cells without affecting noncancerous cells. The physicochemical properties of the nanocomposite hydrogel plus its photothermal capability to trigger the release of 5-FU and ibuprofen drugs by NIR irradiation make the H-MWCNT-f hydrogel attractive for the controlled release of hydrophilic and hydrophobic drugs on demand and treatment requirements.

## ABBREVIATIONS

H-polymer: polymer hydrogel
H-MWCNT-f: nanocomposite hydrogel
MWCNT-f: functionalized multiwalled carbon nanotubes
